# Future directions and priorities in sepsis epidemiology research: a call for action

**DOI:** 10.2471/BLT.20.276709

**Published:** 2021-03-02

**Authors:** Alessandro Cassini, Carolin Fleischmann-Struzek, Mohsen Naghavi, Konrad Reinhart, Benedetta Allegranzi, on behalf of the WHO Sepsis Expert Technical Group

**Affiliations:** a Department of Integrated Health Services, World Health Organization, Avenue Appia 20, 1211 Geneva 27, Switzerland.; b Center for Sepsis Control and Care, Jena University Hospital, Jena, Germany.; c Department of Health Metrics Sciences, University of Washington, Seattle, United States of America.; d Department of Anesthesiology and Operative Intensive Care Medicine, Charité Universitätsmedizin Berlin, Berlin, Germany.

At the Seventieth World Health Assembly held in May 2017, the World Health Organization (WHO) Member States endorsed Resolution WHA70.7 *Improving the prevention, diagnosis and clinical management of sepsis*.^1^ The resolution requested WHO to support Member States in defining standards and establishing the necessary guidelines, infrastructures, laboratory capacity, strategies and tools for reducing the incidence of mortality from and long-term complications of sepsis.

Although sepsis has been mentioned in the medical context since antiquity, it is still a frequent, but preventable, condition. Sepsis is characterized by a dysfunctional host response to infection and is the final common pathway to death from most infectious diseases worldwide. Sepsis incidence and mortality mainly represents the evolution of diarrhoeal diseases and lower respiratory infections, but it is also a common consequence of infectious complications of injuries and noncommunicable diseases,^2^ all morbidities that are high global health priorities.

Understanding sepsis, its epidemiology and burden, remains challenging. While some reports have provided alarming estimates of its global burden,^2^^–^^5^ others have expressed strong concerns about the reliability of sepsis data collection and the appropriateness of attributing deaths to sepsis in patients with multiple comorbidities.^6^ Indeed, high-quality data on the burden of sepsis are limited by the inconsistent application of sepsis definitions, variable diagnostic criteria, few prospective studies with a narrow geographical coverage, and suboptimal availability of administrative data and hospital discharge coding. Despite these challenges, WHO released the first-ever global sepsis report on 9 September 2020, building upon the careful review, analysis and interpretation of existing research on sepsis epidemiology and its burden in different settings and patient populations.^7^ This work also disentangles the methodological approaches and the limitations hampering data comparability, quality and relevance, and identifies critical knowledge gaps.

## Sepsis research complexity

Research on sepsis epidemiology and its burden should ideally rely on prospective studies based upon clinical data from patient records and/or on community-based study designs. However, the feasibility of this approach is hampered by the lack of resources and systems enabling research, among other challenges. Furthermore, sepsis case definitions have changed over time, differ according to age groups, and have limited applicability in low-resource settings, depending on diagnostic capacity. These constraints limit data collection and standardization and introduce significant heterogeneity across studies. Conversely, using clinical criteria, such as the systemic inflammatory response syndrome criteria, might be simpler, but potentially overly sensitive and lacking specificity. Estimations of the epidemiological impact of sepsis have mainly relied on systematic literature reviews that included observational cohort or cross-sectional studies mostly based on hospital coding data for case detection, usually the codes from the International Statistical Classification of Diseases and Related Health Problems (ICD). However, sepsis coding data are inherently biased because sepsis coding is often suboptimal and suffers particularly from low sensitivity compared to the gold standard of clinical sepsis diagnosis in patient charts. Coding may also be influenced by reimbursement incentives in health-care services. Moreover, sepsis deaths might be coded exclusively for their underlying infection. Thus, results based on administrative health-care data as the main source generally represent an underestimation. However, the 11th revision of ICD introduced the possibility to convey patient case-mix and appropriately describe the complexity of multiple causes of disease and death. This new approach represents an opportunity for researchers to improve the analysis of the role of sepsis as a risk factor for death and long-term sequelae.

## Interpreting sepsis estimates

The Institute for Health Metrics and Evaluation global burden of sepsis study (building on the Global Burden of Disease Study) estimated sepsis incidence by modelling sepsis-related case fatality from hospital administrative data, and sepsis-associated mortality using multiple sources of cause-of-death vital registration data.^2^ Although this model represents a significant step forward in providing recent estimates of sepsis cases and deaths, global results were based on data extrapolation for in-hospital case fatality rates from 10 countries (Austria; Brazil; Canada; Chile; Georgia; Italy; Mexico; New Zealand; Philippines; and United States of America) and deaths associated with sepsis from four countries or territories (Brazil; Mexico; Taiwan, China; and United States), none of which was low-income. Thus, the interpretation of epidemiological time trends showing a decrease over the past 3 decades may be unreliable, given that the reduction in upper-middle-income countries was projected to low-income settings.

As expected, both the Institute of Health Metrics and Evaluation methodological approach^2^ and findings from systematic reviews^3^^–^^5^ revealed that the biggest gap of evidence on the burden of sepsis concerns low- and middle-income countries. Low-resource settings also have a higher burden of infectious diseases (estimated to be 85% of global sepsis morbidity and mortality in 2017),^2^ limited infection prevention, and fewer resources for sepsis treatment and intensive care. Improving our understanding of the epidemiology of sepsis in low-resource settings is therefore critical. With these gaps in mind, WHO initiated several partnerships and high-quality studies on the clinical management of sepsis with Alliance for Maternal and Newborn Health Improvement,^8^ African Neonatal Sepsis Trial,^9^^,^^10^ Simplified Antibiotic Therapy Trial,^11^ Global Maternal Sepsis Study,^12^ Multi-Country Survey on Abortion and the Global Antibiotic Research and Development Partnership. These studies provide epidemiological data and a focus on low- and middle-income countries.

Moreover, WHO has established a technical group of international experts to facilitate discussions and consensus on the current status of sepsis epidemiology research and limitations inherent in the methods used to identify sepsis morbidity and its burden. These experts have also worked towards identifying approaches to achieve a better standardization of sepsis epidemiology research and define its future directions and priorities to close existing gaps (Box 1). The expert group identified short- and longer-term priorities at the global level and more specific actions recommended for different settings, according to all available resources.

Box 1 **Summary of international expert consensus on future directions and priorities in sepsis epidemiology research**

**Short-term priorities for sepsis epidemiology research (next 5 years)**

Global

- Advocacy and funding for generating evidence in sepsis epidemiology (all aspects of the burden of disease: etiology; incidence and prevalence; risk factors; outcomes; and the economic impact of sepsis) according to scientific standards for high-quality research at all levels of health and resource settings.
- Achieve international consensus on a global sepsis case definition for sepsis surveillance and epidemiological research, in accordance with the ICD-11 classification, linked to clinical research on sepsis (for example, tiered definitions), specific to relevant age groups and applicable in low- and middle-income countries.
- Promote linkage of sepsis surveillance and epidemiology research to ICD-11 classification coding, clinical data and microbiological results.
- Assess the role of sepsis in existing action plans (e.g. antimicrobial resistance, patient safety, universal health coverage, water, sanitation and hygiene, infection prevention and control, maternal and child health programmes) at all levels (global, regional and national) and advocate for appropriate inclusion of sepsis.
- Develop recommendations on the design and reporting of sepsis epidemiological studies, based on existing tools (for example, Strengthening the Reporting of Observational Studies in Epidemiology checklists).
- Promote research on the linkages between sepsis and other global priorities, such as universal health coverage, quality of care, antimicrobial coverage, infection prevention and control, water, sanitation and hygiene, and maternal and child health.

Low-resource settings

- Support through advocacy, funding and technical assistance, including population-based primary research on sepsis epidemiology (including health facilities and communities) in line and in cooperation with similar existing initiatives (e.g. Global Antimicrobial Resistance and Use Surveillance System).
- Building and strengthening laboratory capacity, in line and in cooperation with similar existing initiatives (e.g. Global Antimicrobial Resistance and Use Surveillance System, research in maternal and child health).
- Promote the linking of results from sepsis epidemiology research to interventions that decrease morbidity and mortality from sepsis, while assessing the feasibility and/or impact of interventions.

**Long-term priorities for sepsis**

- Strengthen evidence on the role of sepsis in high-risk populations (e.g. related to age, underlying disease and vulnerable groups).
- Strengthen evidence on sepsis-causing organism prevalence and antimicrobial susceptibility profiles.
- Prioritize diagnostic and prognostic tests (e.g. biomarkers) for early recognition at bedside testing and to improve clinical outcomes adapted and affordable for low-resource settings.

**Next steps towards a comprehensive global sepsis monitoring**

- Map and assess relevant existing surveillance systems (e.g. Health and Demographic Surveillance System, Global Antimicrobial Resistance and Use Surveillance System; dengue, influenza, malaria, meningitis, tuberculosis); explore and recommend synergies for sepsis surveillance.
- Develop a core set of process, quality and structure indicators to evaluate sepsis prevention and response capacity.
- Build a stakeholder network to reach consensus on sepsis case definitions and best practices for epidemiological surveillance and research, including development of a minimum core data set and appropriate data collection tools.
- Explore the feasibility and resources required to promote a surveillance event (e.g. prevalence study) during World Sepsis Day.

ICD: International Statistical Classification of Diseases and Related Health Problems.Note: Summary based on the outcomes of the Technical Expert Meeting on Methodology for Sepsis Epidemiology Research. Geneva: World Health Organization; 28–30 October 2019.

## Future research

A more complete picture of the impact and prevention of sepsis worldwide requires more evidence on its epidemiology, notably in low- and middle-income countries. In the short term, advocacy and funding of high-quality research in sepsis epidemiology is crucial to ensure global comparability and generate this evidence. Particularly in low-resource settings, these efforts should build on, cooperate and be aligned with similar initiatives, such as the Global Antimicrobial Resistance and Use Surveillance System, including areas such as strengthening of laboratory capacity. For example, the Sequential Organ Failure Assessment score to operationalize the sepsis definition represents a hurdle in health-care settings with limited laboratory services; potentially, an alternative case definition for the purpose of epidemiological studies would be useful. A stepwise or tiered case definition ranging from purely clinical to full laboratory confirmation would increase surveillance feasibility and provide evidence from settings where the burden is highest.

Furthermore, the priorities identified by the WHA70.7 resolution include a call to better estimate the attributable mortality of sepsis and the effects of sepsis on the quality of life of survivors, including identifying risk factors, key drivers and contextual determinants of its epidemiology. Evidence generated through such estimations would represent a clear opportunity for translation into feasible and cost-effective interventions that decrease the burden of sepsis. Information on the quality and completeness of available data sources, and best approaches to integrate these data, would support a coherent approach to the design of studies in sepsis epidemiology. Moreover, a protocol representing a gold standard would be an incentive to adopt the ICD-11 classification to report and analyse multiple causes of death. Globally, a short-term goal should be to integrate elements of sepsis into established programmes (such as antimicrobial resistance) and disease-specific surveillance systems.

Further research in diagnostic and prognostic tests such as biomarkers for early recognition at the bedside, both adapted and affordable for low-resource settings, would enable improved surveillance. In the longer term, such diagnostic and prognostic tests would strengthen the evidence on the organisms causing sepsis, thus informing the local and regional antimicrobial susceptibility profiles, as well as an adapted and tailored clinical management of sepsis cases. Routine surveillance could be initiated in populations at risk (due to age, underlying conditions or being displaced), or according to geographical areas and settings. Intermittent prospective studies are also a low-cost alternative to ongoing surveillance and could provide more evidence on the long-term consequences of sepsis. Integration of sepsis and its role in existing action plans at all levels is also a global priority that should be promoted within other global priorities, such as universal health coverage, quality of care, antimicrobial coverage, infection prevention and control, water, sanitation and hygiene, and maternal and child health.

Given its contribution to preventable mortality and morbidity across different diseases, combating sepsis is an integral part of realizing targets 3.1, 3.2, 3.3 and 3.8 of the health-related sustainable development goal (SDG) 3. Sepsis can also be a significant complication of injuries and noncommunicable diseases, providing another key connection with SDG 3. Together with promoting sepsis prevention and improving its clinical management and diagnosis through early recognition, surveillance is key to better understanding the problem, ultimately contributing to patient safety and quality of care.
